# Baseline Conservative and Surgical Management in the Treatment of NSAID-Exacerbated Respiratory Disease

**DOI:** 10.3389/falgy.2021.659887

**Published:** 2021-05-28

**Authors:** Saara Sillanpää, Jura Numminen

**Affiliations:** Department of Ear and Oral Diseases, Tampere University Hospital, Faculty of Medicine and Health Technology, Tampere University, Tampere, Finland

**Keywords:** chronic rhinosinusitis with nasal polyps, nasal polyps, asthma, aspirin allergy, NSAID-exacerbated respiratory disease

## Abstract

Non-steroidal anti-inflammatory drug (NSAID)-exacerbated respiratory disease (N-ERD) is a chronic medical condition that includes asthma, chronic rhinosinusitis with nasal polyposis, and hypersensitivity to aspirin and other NSAIDs. Eosinophilic inflammation in the upper and lower airways is treated with local corticosteroids, repeated antibiotics, oral corticosteroid courses, endoscopic sinus surgery, and in some cases aspirin treatment after desensitization (ATAD). Nevertheless, the disease may be uncontrolled and it has a great impact on quality of life. A better understanding of the pathomechanisms of the disease and the development of medications that target type 2 inflammation mediators may have a crucial role in achieving better disease control in patients with N-ERD.

## Introduction

NSAID-exacerbated respiratory disease (N-ERD), also known as aspirin-exacerbated respiratory disease (AERD) or Samter's triad, is a chronic eosinophilic inflammatory disorder that is defined as hypersensitivity to NSAIDs together with asthma and chronic rhinosinusitis with nasal polyposis (CRSwNP). Although already clinically described by Samter and Beers (1), the pathomechanism of N-ERD is still only partially understood. It is known, however, that reactions to NSAIDS are a result of the inhibition of the cyclooxygenase enzyme COX-1. Moreover, there is a hypothesis that the inhibition of prostaglandin E2 biosynthesis triggers the activation of inflammatory cells, leading to the release of histamine, cysteinyl leukotrienes, and a cluster of other mediators that are responsible for the clinical symptoms (2). The pathogenesis of chronic extensive eosinophilic mucosal inflammation in the upper and lower airways is related to abnormalities of both the cyclooxygenase- and lipoxygenase-derived arachidonic acid metabolism and the overproduction of Th2-type inflammation mediators (2).

The prevalence of N-ERD varies from 1.8 to 44%, depending on the population observed (3, 4). In patients with adult onset asthma, the prevalence is between 5 and 10% (5). The onset of N-ERD symptoms is most often seen during the 3rd and 4th decade of life (6), and the most important clinical characteristic is eosinophilic CRSwNP. Patients with N-ERD suffer from nasal obstruction, nasal drainage, hyposmia or anosmia, and facial pressure. In addition, CRSwNP is diagnosed with nasal endoscopy and the extent of the disease, i.e., mucosal hyperplasia, can be seen in computed tomography (CT) scans. The polyposis can be graded with the Lund-Kennedy endoscopy score and the extent of the disease from CT scans with the Lund-Mackay CT score. Moreover, the severity of symptoms and the quality of life of these patients can be measured with the sinonasal outcome test (SNOT-22), which is a disease-specific, health-related 22-item quality of life questionnaire.

Upper airway symptoms occur 1-5 years before asthma symptoms (7). In most cases, asthma is usually non-atopic and quite often moderate to severe, and the risk of uncontrolled asthma is two-fold in patients with N-ERD (8). Furthermore, these patients also have an increased risk for difficult exacerbations and hospitalization (8). N-ERD should be suspected if a patient with CRSwNP and asthma, reports upper or lower respiratory symptoms that occur after the intake of NSAIDs. Acute clinical reaction to NSAIDs is usually seen within 30-180 min after the intake. The reaction includes upper airway symptoms such as nasal obstruction and rhinorrhea. These symptoms are then followed by lower airway symptoms, such as wheezing, coughing, and difficulties in breathing. These reactions are essential in the diagnosis of N-ERD. In uncertain cases, a challenge test with aspirin can be performed under controlled conditions (2).

The management of these patients includes medical treatment as well as endoscopic sinus surgery (ESS). Moreover, the treatment of patients is often a collaboration of different specialties, such as an ENT specialist, a pulmonologist, and an allergologist. From the ENT specialist's point of view, these are the most challenging CRSwNP patients who may not respond well to the given treatment and who will need follow-up and regular contacts with their health-care unit.

## Medical Management

The goal of the treatment is to cure the upper and lower airway symptoms, to prevent CRS and asthma exacerbations, to prevent airway remodeling, and to improve the patients' quality of life. The first treatment for NSAID hypersensitivity is the avoidance of these drugs. Therefore, patient education is crucial. Pharmacological therapies for CRSwNP and asthma follow general national and international guidelines that focus on the underlying mucosal eosinophilic inflammation of the upper and lower respiratory tracts (9, 10).

The medical treatment of CRSwNP in patients with N-ERD is similar to that in patients who are NSAID tolerant (9). CRSwNP symptoms are treated with daily nasal saline irrigations, regular intranasal corticosteroids sprays or drops, short-term oral corticosteroids (OCS) in cases where maximal doses of intranasal corticosteroids are not able to control the severity of CRS, and short-term antibiotics when bacterial infection is suspected. OCS are especially effective in reducing symptoms and polyp size and are used as a rescue treatment in CRS exacerbations (9). OCS are also used in the control of lower airway symptoms. Indeed, one study reported that 32% of patients with N-ERD used systemic steroids daily and that 45% needed shorter courses (6). However, the use of OCS is not an optimal long-term therapy due to the known adverse effects, such as the risk of osteoporosis, gastrointestinal symptoms, hypertension, and metabolic disorders (11).

Patients with N-ERD have an overproduction of cysteinyl leukotrienes and it seems that the leukotriene-modifying drugs (LTMD), Montelukast or Zileuton, could be a rational treatment of choice for patients with N-ERD. Studies have shown their efficacy in asthma and allergy symptoms (12–14), but Montelukast was not superior in patients with N-ERD when compared to patients who are NSAID tolerant, as both study groups showed similar improvements in the parameters studied in a single-blind, placebo-controlled study (15). LTMD have a moderate effect in relieving nasal symptoms, but their effect may not be more effective in patients with N-ERD than in patients who are NSAID tolerant (16). Further, their effect is inferior to intranasal corticosteroids (16) and international guidelines do no recommend LTMD as an add-on treatment in patients with CRSwNP (2, 9). However, if patient with N-ERD is taking LTMD due to asthma or allergy, it is recommended to continue the medication especially during aspirin challenge or ATAD to provide stability of the airways (17).

The treatment of asthma depends on the severity of the symptoms, and a standard step-wise approach is used until the asthma is under control (10). Treatment consists of a combination therapy with inhaled corticosteroids and long-acting beta-2 agonist, and OCS to control exacerbations. Patients who have difficult-to-treat asthma can benefit from new biological treatments, such as omalizumab, dupilumab, and mepolizumab (10). Recent studies on these monoclonal antibodies have also reported promising results in both symptom relief and/or polyp formation in patients with CRSwNP (18–21). These new agents may also provide substantial benefit in the treatment of resistant nasal polyposis in patients with N-ERD.

## Surgical Treatment

ESS is essential in the management of upper airway disease. The goal of surgical management is to remove hyperplastic and polypoid tissue from the nasal cavity and sinuses. This results in improved sinus drainage, better exposure for topical drug delivery, and decreased inflammatory load. The aim of the surgery is always to improve the patients' symptoms, and if the patient is uncontrolled with appropriate medical treatment and there are obvious endoscopic and CT scan findings, surgical treatment is recommended. Moreover, a pre-operative SNOT-22 score of more than 20 points indicates that a patient is likely to benefit from surgery, as the pre-operative symptom score is the best predictor of the success of surgery and those with higher symptom scores preoperatively achieve greater improvement after surgery (9). However, surgical treatment alone is not effective, and it is therefore always used in combination with long-term medical treatment.

The extent of surgical intervention varies from patient to patient. The severity of nasal polyposis is evaluated in pre-operative CT scans. Often, patients with N-ERD present extensive sinus opacification and have very high Lund-Mackay CT scores (22). In these cases, extended surgery is needed due to an aggressive inflammatory process of the mucosa. Complete sinus opening includes anterior and posterior ethmoidectomies, middle meatal antrostomy, shpenoidotomy, and frontal sinus opening bilaterally. It has been shown that extended ESS reduces symptom scores among patients with CRS (23) and the need for revision surgery compared with partial ESS (24). A recent study has shown that partial ethmoidectomy in patients with N-ERD increased the risk of revision surgery (25). Furthermore, recurrent ESS surgeries predicted uncontrolled N-ERD (25). ESS reduces patients' symptoms reported in the SNOT-22 scores, and this effect is also objectively seen in improvement in nasal endoscopy and CT scans postoperatively in patients with N-ERD (26). The short-term effects of ESS in patients with N-ERD are comparable to patients with CRSwNP overall, but patients with N-ERD need revision surgery earlier in the post-operative period (27) and these patients undergo two-fold more sinus surgeries at a younger age compared to NSAID-tolerant patients with CRSwNP (28).

## Aspirin Treatment After Desensitization

Aspirin treatment after desensitization (ATAD) can be recommended if the patient's disease is uncontrolled despite repeated ESS surgeries and the adequate medical management of asthma and CRSwNP. Indications for ATAD are low response to other pharmacological treatments, fast recurrence of nasal polyposis after surgery, difficult-to-treat asthma, need to reduce the dose of oral corticosteroids, or other indications of the need for anti-inflammatory drugs, such as rheumatoid arthritis. The optimal aspirin dosing, duration of the desensitization, or indications have not been validated in randomized, placebo-controlled studies and there are several protocols for how to perform ATAD. At present, however, it seems that the effective oral maintenance dose of aspirin ranges from 100 to 1,300 mg (29–31).

ATAD results in a significant clinically relevant reduction in SNOT scores. A meta-analysis of three double-blinded randomized and placebo controlled trials showed 11.9 points reduction in SNOT-22 scores (29, 32, 33). ATAD treatment can also decrease the burden of repeated surgical operations and the adverse effects of repeated OCS treatments (34, 35). In a study by Berges-Gimeno et al. 14% of patients discontinued the treatment due to adverse effects and 11% due to poor response or other reason. Finally, 64% of the study patients who continued the treatment after 1 year benefitted from the treatment (36). The most common adverse effects of ATAD are gastrointestinal pain and bleeding or non-gastrointestinal bleeding, such as epistaxis (36). To minimize the gastrointestinal risks, proton pump inhibitors or H2-blockers are recommended during the treatment and *Helicobacter pylori* should be tested and eradicated if present before initiation of the treatment (2). However, the ATAD discontinuation rate might be as high as (63%) due to side effects or weak response (37).

It is recommended that ESS is performed prior to aspirin desensitization to minimize polyp regrowth during ATAD treatment. Studies have shown that ESS performed prior to ATAD reduces the need for revision surgery from an average of once every 3 years to once every 10 years (34, 35). Patients with higher baseline serum IgE levels especially benefited from ESS performed shortly before ATAD (38). The combination of ESS and ATAD also decreased overall topical corticosteroid and OSC use (38, 39).

## Discussion

The treatment of patients with N-ERD is challenging and the pathomechanism still remains partly unknown. Moreover, N-ERD is a long-lasting disease, and therefore patients should have regular contact with their nursing health-care unit. It is also of the utmost importance that patients are encouraged to use conservative treatment for their asthma and CRSwNP regularly and that patients are informed that the treatment should be continued despite surgical treatment. From a surgical point of view, it seems to be important that extensive initial surgery is performed for patients with N-ERD. Better disease control may be achieved if complete ESS is performed as a first-line treatment, and it may also reduce the need for revision surgery. Often, revision surgery is riskier than the initial surgery due to the absence of anatomical landmarks. In a review article, Muhonen et al. (40) discussed the role of the surgery in patients with N-ERD and pointed out that without surgery many sinuses may remain inaccessible to saline irrigation and topical drug delivery. Therefore, an appropriate extent of surgery should provide large enough ostia to the sinuses. From this point of view, they suggested that Draf III frontal sinusotomy may be an effective early intervention in patients with N-ERD (40). Still, more studies on the extent of optimal initial ESS surgery are needed in this patient group, and the current literature lacks strong guidelines.

Patients with N-ERD do not always reach an adequate level of control despite being given appropriate treatment. A patient with difficult-to-treat CRS is defined in the European Position Paper of Rhinosinusitis and Nasal Polyps 2020 as a patient who has received adequate surgical treatment, intranasal corticosteroid treatment, and two short courses of antibiotics or corticosteroids in the last year (9). Many patients with N-ERD still belong to this group and have poor quality of life. ATAD has been recognized as an accepted therapeutic modality that should be considered in this patient group. Indeed, in patients with good response to ATAD, a better control of asthma and CRS symptoms can be achieved (30). ATAD is meant to be long-lasting treatment and to prevent allergic reactions aspirin should be taken strictly every day. However, several unanswered questions regarding ATAD, such as the optimal dosage and initiation time of the treatment, still remain. The side effects of ATAD also limit the use of this treatment option. Some major complications, such as clinically significant gastrointestinal bleeding and anaphylaxis, have been reported in ATAD treatment. Other minor reasons for the discontinuation of the treatment include gastritis, persistent sinonasal symptoms, recurrent epistaxis, and asthma exacerbations (41). Not all patients tolerate or benefit from ATAD. In the literature, the discontinuation rates vary greatly from 16 to 63% (36, 37, 41), and therefore alternative treatment options are needed.

N-ERD has been considered as a separate asthma phenotype, but it can be divided into several sub-phenotypes with variable clinical characteristics. A study classified patients with N-ERD depending on asthma control and severity, intensity of upper airway symptoms, severity of airway obstruction, and health-care use and found four different sub-phenotypes (42). These patients also differed in blood eosinophilia and urinary leukotriene E4 levels, and those patients with intensive upper airway symptoms had the highest levels of these markers (42). Heterogeneity in lower airway inflammation mediators has also been detected in patients with N-ERD, since a part of them had more changes in eicosanoid profile and others more changes in Th2-type inflammation profile (43). There is also increasing evidence that N-ERD is a heterogenous disease with different pathomechanisms, i.e., sub-endotypes in the background (44).

Not all patients with N-ERD respond to the basic medical and surgical management discussed above. These different sub-endotypes of the disease could serve to explain the variable responses to different treatments (45). Patients with N-ERD are the most difficult to treat in the CRSwNP patient population and this is also reflected on the cellular and molecular level as increased levels of the inflammatory mediators (46). The new biological agents that target the inflammation mediators could be good alternatives in the treatment of uncontrolled patients. Some studies have focused on the effect of these biological agents, especially in patients with N-ERD. The effects of Omalizumab, which is an anti-IgE antibody, were studied by Jean et al. in patients with N-ERD. They found that Omalizumab decreased yearly OCS use (47). In addition, Hayashi et al. (48) showed that daily OCS dose and nasal symptom scores decreased with Omalizumab in patients with N-ERD. Mebolizumab is an IL-5 antagonist which reduces eosinophilia. Mebolizumab could also be beneficial in patients with N-ERD since a significant reduction in daily OSC dose and SNOT-22 scores and a decrease in absolute eosinophil counts were seen in a retrospective study that consisted of 14 patients (49). Dupilumab is an IL-4 and IL-13 antagonist and both of these cytokines induce local IgE production. Furthermore, IL-13 has other effects, for example, on the epithelial differentiation causing mucus secretion and fibrosis. Laidlaw et al. (50) analyzed the effect of Dupilumab in a subgroup of 19 patients with N-ERD from a cohort of a phase 2 Dupilumab trial. The results clearly demonstrated that patients with N-ERD benefitted from Dupilumab. It reduced Total Polyp Score by 2.5 points and SNOT-22 score by 30 points. Patients also had a clear improvement in smell identification test (50). However, the number of the patients in each of these studies was quite low (from 14 to 29) and the length of the therapy was relatively short (between 3 and 12 months) (47–50). Therefore, there is a need for longer lasting studies in larger N-ERD patient groups.

Patients with N-ERD have reduced quality of life and require frequent medical and surgical treatment for recalcitrant disease. The development of new biological agents that target the specific and critical pathways of the pathogenesis of the disease could be an alternative treatment option in uncontrolled patients. Also, specific biomarkers in the endotyping of patients could help in the planning of more individualized treatments. There have already been some positive results in the treatment with monoclonal antibody medications of patients with N-ERD (47–50) and even more in overall CRSwNP patients (18–21). So far, Dupilumab is the only monoclonal antibody approved for the treatment of CRSwNP. However, the cost of the treatment and the lack of long-lasting results limits its use in the wider CRSwNP population. As this treatment is presently available, it is logical that this expensive treatment is offered to the most difficult, uncontrolled patient group. In the CRSwNP population, this often means patients with N-ERD. In future, these biological treatments may become more widely used, and then the role of ATAD may be re-evaluated.

## Author Contributions

SS and JN contributed to conception and design of the study and wrote the first draft of the manuscript together. JN wrote the sections introduction, ATAD, and discussion of the manuscript. SS wrote the sections surgical and conservative therapy and discussion of the manuscript. All authors contributed to manuscript revision, read, and approved the submitted version.

## Conflict of Interest

The authors declare that the research was conducted in the absence of any commercial or financial relationships that could be construed as a potential conflict of interest.
